# Hydrogeochemical Processes and Connection of Multi-Layer Groundwater System in Sunan Mining Area, Eastern China

**DOI:** 10.3390/ijerph191912392

**Published:** 2022-09-29

**Authors:** Qiding Ju, Youbiao Hu, Kai Chen, Qimeng Liu

**Affiliations:** 1 State Key Laboratory of Mining Response and Disaster Prevention and Control in Deep Coal Mines, Anhui University of Science and Technology, Huainan 232001, China; 2 School of Earth and Environment, Anhui University of Science and Technology, Huainan 232001, China

**Keywords:** mining area, hydrogeochemical, reverse model, multi-layer groundwater, multivariate statistics, APFS-MLR model

## Abstract

Groundwater is an important freshwater resource in the world and serves as the main source of water for mining areas in Northern China. Coal mining may cause changes in water quality. As such, to identify ways to prevent water contamination, this study investigates the hydrogeochemical processes and transport paths of a complex aquifer system in the Sunan mining area in Northern China. Using the APFS-MLR model, a geographic information system (GIS) spatial analysis, and a hydrochemical correlation analysis method, this study identifies the potential mineral phases in groundwater, the spatial distribution of mineral reactions, and the contribution rate of these reactions to hydrochemical variables. Inverse modeling is used to verify hydrogeochemical process. The study reveals the relationship between multiple aquifers and four hydrological transport paths. Here, Path 1 and Path 2 show that the Quaternary aquifer, Carboniferous aquifer, and Ordovician aquifer are recharging the Permian aquifer through mineral dissolution and precipitation, cation exchange, and sulfate reduction. On the other hand, Path 3 and Path 4 show that tthe connections of Carboniferous and Ordovician limestone aquifers are dominated by the dissolution and precipitation of minerals and cation exchange, and that they are mainly recharged by the Quaternary aquifer. In the future, the water level of the Permian aquifer may rise somewhat after mining ends, and the mixing of water from the Permian aquifer, Quaternary aquifer, Carboniferous aquifer, and Ordovician aquifer could cause cross-pollution. In addition, sewage produced by human activities may recharge the deep water through the shallow water, polluting the deep karst water. As such, measures should be taken to reduce the hydraulic connection between Permian mine water and karst aquifers. The results of this study may benefit water quality predictions and treatment approaches in other complex multi-layer aquifer areas in the world.

## 1. Introduction

Although many forms of new energy are emerging in China, coal resources continue to dominate China’s energy structure system [1,2]. However, extracting coal resources can induce instability in underground multi-layer aquifer systems, leading to changes in the underground hydrogeochemical environment and allowing the inrush of coal mine roadway water. This can seriously threaten groundwater quality and coal mine safety [3,4,5,6]. In addition, groundwater is an important source of water in Northern China [7]. According to statistics, urban domestic and industrial water use accounts for 80–90% of total groundwater use, and agricultural water use accounts for about 10–20%. This highlights the importance of clarifying the hydrogeochemical mechanisms at play in multi-layer aquifer systems before harnessing and protecting the groundwater environment [8,9,10,11,12,13].

Scholars have conducted many groundwater research studies in cities [14,15,16], coal mines [17,18,19], and basins and plains [20,21,22]. These studies mainly focus on groundwater circulation [23], groundwater resource evaluations [24,25], groundwater quality evaluations [26,27], and the evolution of the groundwater hydrogeochemical environment [28,29]. These studies have mostly analyzed a specific aquifer. Few studies have been conducted on multi-layer aquifer systems in mining conditions. There have also been few analyses of the contribution of the main factors controlling the composition of water chemistry. This highlight current gaps in the systematic research about the mechanism by which hydrogeochemistry forms in a multi-layer groundwater system.

Given these challenges, research methods are becoming increasingly mature, and include testing and analyzing ion components in water samples, as well as conducting statistical analyses of the results. The main methods currently applied include hydrochemical type analysis, isotope analysis, simple scatter plot analysis, correlation analysis among ion components, saturation index calculations, multivariate statistical analysis, and hydrogeochemical simulations [30,31,32,33,34]. Good results have been achieved in studies analyzing the hydrochemical characteristics of mining areas and groundwater sources, the hydrochemical evolution process, and water quality and water quality. The studies have also investigated groundwater migration paths and the recharge and discharge characteristics between groundwater aquifers. Therefore, comprehensively applying different methods can reasonably and comprehensively explain the hydrogeochemical genetic mechanisms shaping multi-layer aquifer systems in mining areas.

**Figure 1:**
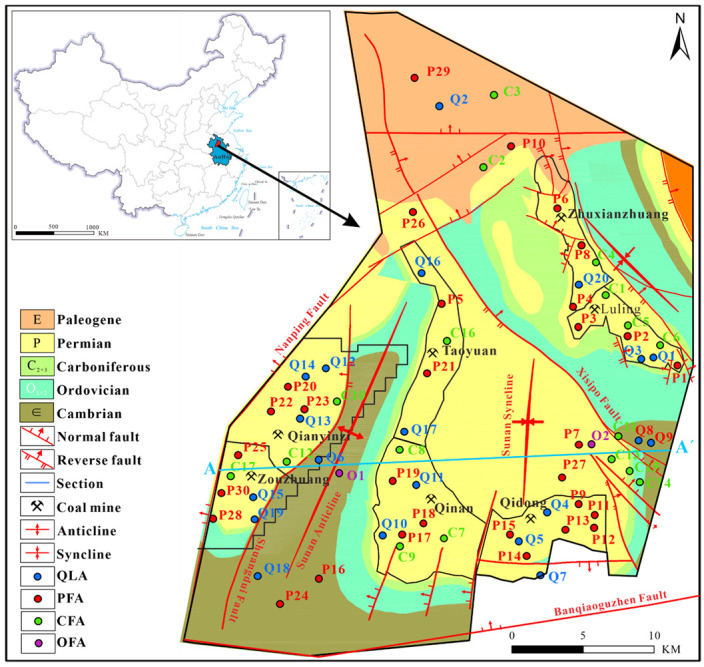
General geological map of the study area and sampling sites (QLA, PFA, CFA, and OFA represent the Quaternary loose aquifer, Permian fracture sandstone aquifer, Carboniferous fracture limestone aquifer, and fracture limestone aquifer, respectively).

The study focuses on the Sunan mining area in the Huaibei Plain, China, which includes seven active coal mines (Figure 1). The shallow groundwater in this area is mainly used for agricultural irrigation and to provide drinking water for the area’s few residents. Previous research on the Sunan mining area mainly focused on shallow water and deep water [35]. For example, Chen et al. (2011) determined the hydrogeochemical characteristics of a deep multi-layer aquifer system in the Sunan mining area using isotope and conventional ion analysis [36]. Wang et al. (2019) studied the hydrochemical characteristics of shallow groundwater in the Sunan mining area, by using conventional hydrogeochemical data and stable isotopes of hydrogen and oxygen [37]. They also determined the main hydrogeochemical processes and formation mechanism. However, few research studies in this area have analyzed the genesis and contribution rate of different hydrogeochemical processes and the transport paths of multi-layer aquifer systems.

To identify the hydrogeochemical processes at work in a multi-layer aquifer system, this paper first studies the source’s contribution of solutes to the multiple layers of the groundwater system. It then analyzes the spatial distribution of hydrogeochemical effects on the multi-layer groundwater system, and assesses the connections between aquifers to determine the connections between the multiple groundwater layers. The study provides a basis for preventing and controlling deep-seated water pollution in the future.

## 2. Study Area

The Sunan mine area is located in the southern part of the Yellow Huai Plain in Northern Anhui Province, China. It is part of the Southeast Secondary Hydrogeological Unit of the Huaibei Coalfield. The study area covers an area of about 330.12 km^2^ and has a resident population of approximately 660,000 people. This area includes the following seven active coal mines: Zhuxianzhuang, Luling Mine, Qidong, Qinan, Taoyuan, Qianyingzi, and Zouzhuang. Permian coal seams are mineable in this region and serve as key coal seams in the entire Huaibei coalfield. The Sunan mining area is located in the middle of the Huaibei Plain, with an elevation of between +23.5 and +24.5 m. The average annual precipitation is between 750 and 900 mm.

**Figure 2:**
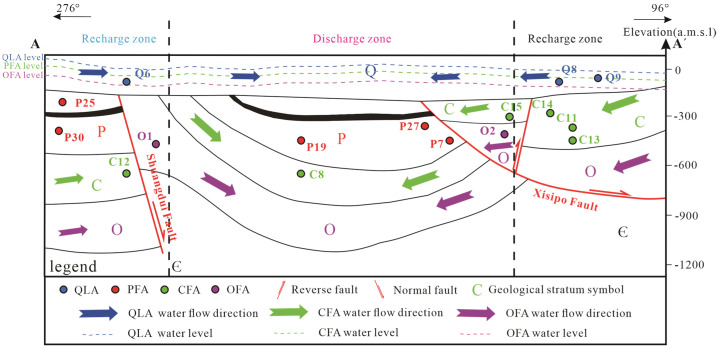
Cross section A–A’ and hydrogeological map of the mining area (Note: Some sampling points are projected positions, see Figure 1).

The southern part of the study area is bounded by the Banqiaoguzhen fault, the northern part is bounded by the Subei fault, and the western part is bounded by the Nanping fault. The area was affected by multi-phase tectonic movement during the Yanshanian period. As such, faults and fissures have developed widely across the study area, possibly connecting different aquifers. According to water level observations in the study area aquifer, the hydrogeological section in Figure 2 shows that the water levels of the Quaternary (Q), Permian (P), Carboniferous (C), and Ordovician (O) aquifers converge from the faults on both sides to the middle syncline. The C and O aquifers are exposed to mining operations, and the replenishment of mine water through faults or fissures creates an important safety hazard for mine production. To simplify the problem, four representative aquifers are selected for study, namely the Quaternary loose aquifer (QLA), Permian fracture sandstone aquifer (PFA), Carboniferous fracture limestone aquifer (CFA), and Ordovician fracture limestone aquifer (OFA). These aquifers are common water inrush aquifers in mining, and they are connected by faults or mining fractures [35,38]. The western part of the profile is the water barrier boundary, and the eastern part is the recharge area (Figure 2). Surface water and groundwater flowed from the high ground to the concave areas before coal mining.

In mining areas, groundwater has been impacted by prolonged human activities, mainly because of the continuous input of large amounts of contaminants into the multi-aquifer system from mining activities. Under normal circumstances, QLA has no direct hydraulic connection with the lower aquifer, and will be connected with PFA under the action of faults and mining fractures [39]. Furthermore, CFA and OFA will connect with QLA in the concealed outcrop area at the angular unconformity [40]. The QLA is composed of sand, gravel and, at the bottom, a clay layer. The sediments mainly consist of silicate and carbonate minerals, and gypsum [41]. The PFA is composed of sandstone, mudstone, siltstone, clay and coal seams, and the groundwater in this formation is mainly stored in the fractures of sandstone layers with static reserves, which is dominated by geological conditions and has various water yield [39]. The CFA includes limestone, mudstone, sandstone, siltstone, clay, and a thin coal seam, and contains unevenly distributed pyrite nodules [42]. The OFA is composed of clay and limestone with a minor amount of pyrite nodules at the top [43].

## 3. Materials and Methods

Water samples were collected from seven mines in the Sunan mining area between 2017 and 2020. A total of 67 samples were collected from multiple aquifers. The location is shown in Figure 1. Some samples were collected from the four aquifers (QLA, PFA, CFA, and OFA) in the mining area. Some samples were collected from the periphery of the mining area, including 20 QLA water samples, 30 PFA water samples, and 17 CFA water samples. The study focused on the QLA, PFA, and CFA aquifers that threaten the mine safety, while OFA is not the main mine water inrush aquifer, as it is controlled by practical factors. The OFA is buried at a depth of nearly 1000 m, and it is very difficult and expensive to obtain water sample data, so only two OFA water samples were collected. Field tests and indoor experiments were conducted to test the water parameters. A portable tester was mainly used to measure pH. Before sampling, clean 500 mL bottles were washed with ultrapure water more than 10 times; the bottles were tightly sealed with bottle caps cleaned with pure water [13,16,44]. The samples were filtered through a 0.45 micron filter membrane. The samples were stored in a low temperature environment to prevent the water quality from changing at higher temperatures [13].

The laboratory measurement were conducted in the water quality testing center of the Anhui University of Science and Technology. The solutes (or components) measured were K^+^ + Na^+^, Ca^2+^, Mg^2+^, SO_4_^2−^, HCO_3_^−^, Cl^−^, and total dissolved solids (TDS). To analyze the cations, samples were acidified with nitric acid to a pH less than 2. The concentration of cations was analyzed using inductively coupled plasma (ICP) atomic emission spectrometry, and the anion concentration was analyzed using ion chromatography. Potassium concentrations were low, so the values were combined with the measurements of the sodium ion. Major ions of all water samples were analyzed within 24 h of collection. The saturation index of calcite, dolomite, and gypsum, and the charge balance value of the water samples, were calculated using PHREEQC 3.6 software. All water samples maintained a charge balance, with error < 5%. The measurement results are shown in Table 1.

This study analyzes large-scale data sets using the Pearson correlation coefficient method, absolute factor analysis method, and the multiple linear regression method. The Pearson correlation coefficient method determines the linear correlation between data variables and can preliminarily explain the factors influencing the main variables in a data set. Factor analysis reduces the dimension of a complex data set by separating irrelevant factors from initial variables. Based on a factor analysis, an absolute principal factor value is introduced. Then, multiple linear regression is used to study the linear relationship between variables and the absolute principal factors. The model quantitatively analyzes the contribution rate of principal factors to variables.

The Pearson correlation analysis was completed using Origin 2021, Learning version. The factor analysis was conducted using the SPSS 26.0 statistical analysis software, and the spatial distribution of the factor score value was analyzed using ArcGIS software. In this study, the major ions of all groundwater samples were interpolated using the Kriging interpolation method. The percent variation (*PV*) between the measured and predicted values was calculated using Equation (1). The sample points were interpolated with the same method to better estimate the areas with a higher or lower *PV*, as follows:

(1) $$PV\left(\%\right)=\frac{V_{p}-V_{m}}{V_{M}}\cdot 100$$

The absolute principal factor multivariate linear regression model (APFS-MLR) was introduced to study the contribution rate of the main factor(s) to each variable. The normalized factor scores and eigenvectors obtained in the factor analysis were used to perform APFS-MLR to quantitatively apportion the sources [14,45]. Details of the APFS-MLR model are described in Thurston and Spengler (1985) [46]. The main steps of this method are as follows:

The original data *X_ij_* is standardized to obtain *Z_ij_*. The factor score *(A_m_)_ij_* of each sample i is calculated as follows:

(2) $$Z_{ij}=\frac{X_{ij}-\bar{X_{j}}}{S_{j}},{\left(A_{m}\right)}_{ij}=\sum_{j=1}^{j}\omega_{j}\cdot Z_{ij}$$

A sample *Z*_0_*_j_* with a zero-concentration value of each variable is introduced and normalized with all samples to obtain *Z*_0_*_j_*. The score *(A*_0_*_m_*)_0_*j* of the zero sample is calculated as follows:

(3) $$Z_{0j}=\frac{-\bar{X_{0j}}}{S_{0j}},{\left(A_{0m}\right)}_{0j}=\sum_{j=1}^{j}\omega_{0j}\cdot Z_{0j}$$

The absolute main factor score APFSmi is calculated as the main factor score *(A_m_)_ij_* of each sample, minus the main factor score *(A*_0_*_m_)_0_j* of the zero sample, as follows:

(4) $${\mathrm{APFS}}_{mi}={\left(A_{m}\right)}_{ij}-{\left(A_{0m}\right)}_{0j}$$

Multiple linear regression is performed with APFS as the independent variable and sample concentration as the dependent variable. The source contribution rate of each ion variable is then calculated using a regression coefficient, as follows:

(5) $$X_{ij}=b_{i}+\sum_{m=1}^{m}b_{mj}\cdot {\mathrm{APFS}}_{mi}$$

(6) $$P_{j}=\frac{b_{mj}}{\sum_{m=1}^{m}b_{mj}}\times 100\%$$

where variable $X_{ij}$ is the initial concentration of the sample, $\bar{X_{j}}$ is the average concentration of variable *j*, and $S_{j}$ is the standard deviation. The variable $\omega_{j}$ is the factor score coefficient, $S_{0j}$ is the standard deviation of the zero-concentration sample, $\omega_{0j}$ is the factor score coefficient when the zero-concentration sample is included, $b_{i}$ is the constant term in the multiple linear regression equation, and $b_{mj}$ is the regression coefficient of the main factor to variable *j*. The variable $P_{j}$ is the contribution rate of main factor m to the *j* variable.

## 4. Results and Discussions

### 4.1. Statistical Analysis

Table 2 shows the statistical results of water samples from the Quaternary loose aquifer (QLA), Permian fracture sandstone aquifer (PFA), and Carboniferous limestone aquifer (CFA). The analysis shows that the pH value of QLA water and CFA water ranges between 6.88 and 9.18, and the pH value of PFA water ranges between 5.59 and 10.96. The entire water unit is weakly alkaline. In this environment, the CO_3_^2−^ levels are far lower than 5% of the total CO_3_^2−^ and HCO_3_^−^ levels when combined. Therefore, CO_3_^2−^ is not included in the component analysis. The statistical results of each aquifer indicate that TDS ranges from 1337–3457 mg/L in the QLA water, with an average of 2017.1 mg/L. The average concentration of cations is greatest for K^+^ + Na^+^, followed in descending order by Ca^2+^ and then Mg^2+^. The average concentration of anions is greatest for SO_4_^2−^, followed by HCO_3_^−^ and Cl^−^. In the PFA water, TDS ranges from 999–4438 mg/L, with an average of 1531.2 mg/L. The order of average cation concentration is the same as with the loose water. The average anion concentration is highest for HCO_3_^−^, followed by SO_4_^2−^ and Cl^−^. In the Carboniferous limestone water, TDS ranges from 904.5–2376.13 mg/L, with an average of 1616.57 mg/L. The order of the average cation and anion concentrations is the same as with loose water. The skewness of each aquifer is close to 0, which conforms to a normal stable distribution. This result indicates that the ion concentrations are relatively stable, due to low interference from external factors and hydrogeological conditions.

### 4.2. General Hydrochemical Analysis

**Figure 3:**
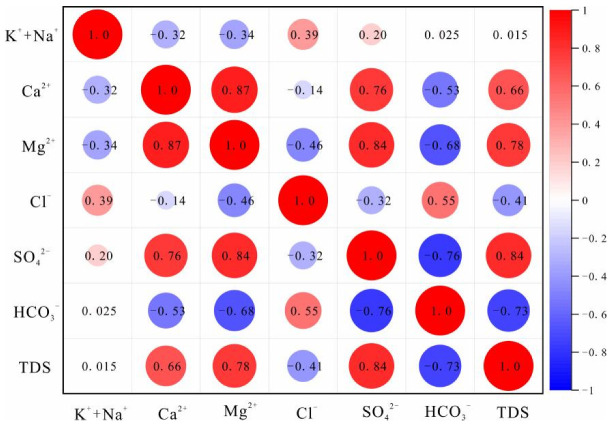
Correlation heat plot for the Quaternary aquifer.

**Figure 4:**
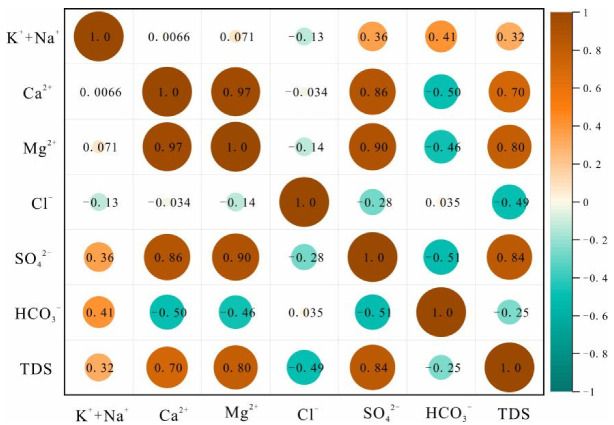
Correlation heat plot for the Permian aquifer.

**Figure 5:**
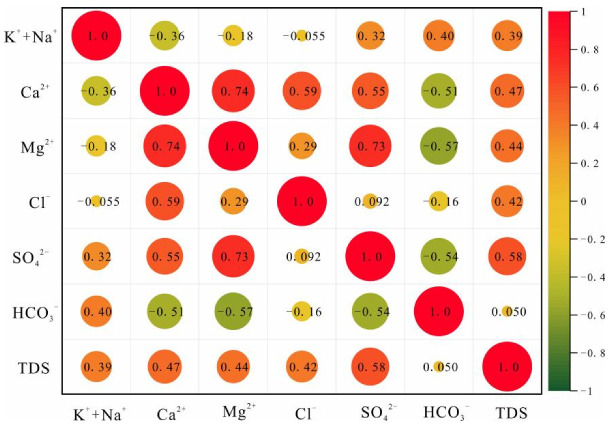
Correlation heat plot for Carboniferous aquifer.

The correlation coefficient heat map of ion concentrations in each aquifer is shown in Figure 3, Figure 4 and Figure 5. The correlations between water sample variables provide a preliminary indication of the consistency of their sources, and whether the same hydrogeochemical process is occurring or has occurred. The results of Figure 3 show that SO_4_^2−^, Ca^2+^, and Mg^2+^ are positively correlated; Cl^−^, HCO_3_^−^ and K^+^ + Na^+^ are weakly correlated; Ca^2+^, Mg^2+^, and HCO_3_^−^ are negatively correlated. This indicates that the dissolution of sulfates, salts, and carbonates are the main water rock interactions in QLA water. It also indicates that Cl^−^ and HCO_3_^−^ may be from the same source. The results of Figure 4 show that SO_4_^2−^, Ca^2+^, and Mg^2+^ are significantly positively correlated, that they are weakly negatively correlated with Cl^−^, HCO_3_^−^, HCO_3_^−^, and K^+^ + Na^+^, and that they are negatively correlated with Ca^2+^, Mg^2+^, and SO_4_^2−^. This indicates that sulfate and carbonate dissolution mainly occur in PFA water. Figure 5 shows that SO_4_^2−^ and Ca^2+^, Mg^2+^, and K^+^ + Na^+^ are positively correlated, and negatively correlated with HCO_3_; HCO_3_ is positively correlated with K^+^ + Na^+^, and negatively correlated with Ca^2+^, Mg^2+^, SO_4_^2−^, and Cl^−^ is positively correlated with Ca^2+^ and Mg^2+^. This indicates that the sulfate dissolution and carbonate dissolution have mainly occurred in the CFA water, and Cl^−^ is consistent as the possible source of SO_4_^2−^.

**Figure 6:**
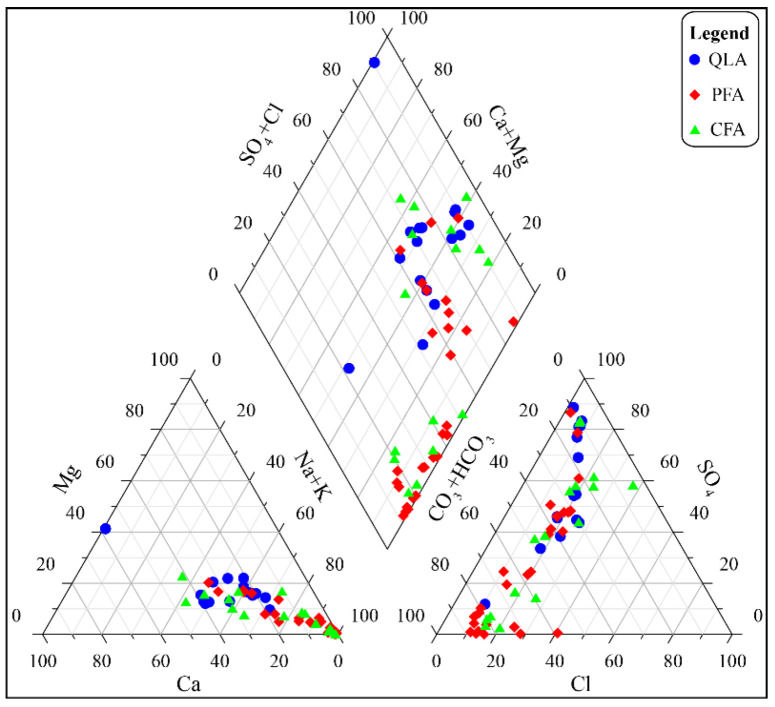
Piper diagram of all water samples.

The Piper trilinear diagram showing the water chemical composition of each aquifer in the mining area is shown in Figure 6. According to the Shukarev classification principle (25% as the boundary), the dominant cation and anion ions of most water sample points in the QLA water are SO_4_^2−^ and K^+^ + Na^+^. A few water sample points are HCO_3_^−^ and Ca^2+^ types. Therefore, the main hydrochemical types of QLA water are SO_4_–Na, HCO_3_–Na, and HCO_3_–Ca. Additionally, K^+^ + Na^+^ is dominant in groundwater. The dominant cation and anion in the PFA water are HCO_3_^−^ and Na^+^, respectively, and the dominant anions in a few water samples are SO_4_^2−^ and Cl^−^. This indicates that the PFA water is mainly of the HCO_3_–Na hydrochemical type. The dominant anions and cations in CFA water are similar to those in PFA water, and the hydrochemical types are mainly HCO_3_–Na and SO_4_– Na. The QLA samples are mainly located in the runoff area, and the PFA water and CFA water are mainly located in the discharge area.

### 4.3. Evidence of Major Hydrogeochemical Processes

#### 4.3.1. Ion Source Analysis

**Figure 7:**
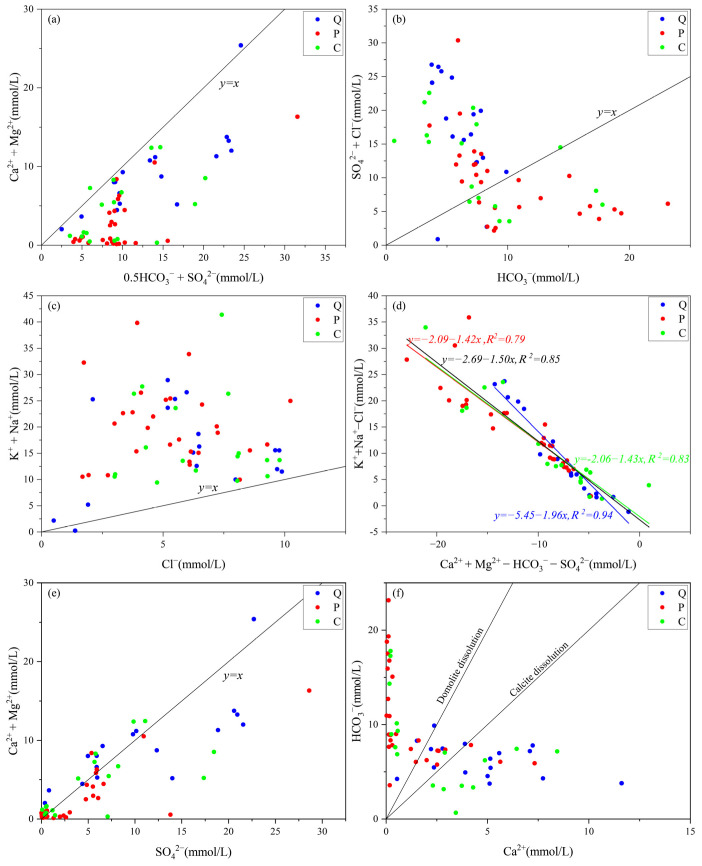
Scatter plots of major ions: (**a**) (Ca^2+^+Mg^2+^) versus (SO_4_^2−^+0.5HCO_3_^−^), (**b**) (SO_4_^2−^ + Cl^−^) versus HCO_3_^−^, (**c**) (K^+^+Na^+^) versus Cl^−^, (**d**) (Ca^2+^+Mg^2+^−SO_4_^2−^−HCO_3_^−^) versus (K^+^+Na^+^ − Cl^−^), (**e**) (Ca^2+^+Mg^2+^) versus SO_4_^2−^, (**f**) HCO_3_^−^ versus Ca^2+^.

In Figure 7a, the ratio of Ca^2+^ + Mg^2+^ to 0.5HCO_3_^−^ + SO_4_^2−^ reflects the source of Ca^2+^ and Mg^2+^ in the aquifer. The ratio in each aquifer is less than 1, so Ca^2+^ and Mg^2+^ mainly come from the dissolution of sulfate rock and carbonate rock. In Figure 7b, the ratios of SO_4_^2−^ + Cl^−^ to HCO_3_^−^ in the QLA water all exceed 1, indicating that the dissolution of evaporites is the main driver. The ratios of SO_4_^2−^ + Cl^−^ to HCO_3_^−^ in a few sandstone water and limestone water samples also exceed 1, indicating that the dissolution of evaporites also occurs. The ratios of SO_4_^2−^ + Cl^−^ to HCO_3_^−^ in other sandstone water and limestone water samples are less than 1, indicating that the dissolution of carbonates also occurs. In Figure 7c, the molar ratio of K^+^ + Na^+^ to Cl^−^ exceeds 1, and the ratio of a few QLA water samples is close to 1. This indicates that sulfate rock and carbonate rock dissolution and cation exchange may have occurred in the Quaternary aquifer, Permian aquifer, and Carboniferous aquifer.

In Figure 7d, the ratio relationship between Na^+^–Cl^−^ and Ca^2+^ + Mg^2+^–SO_4_^2−^–HCO_3_^−^ can be used to determine whether there is cation exchange in the aquifers. The negative correlation between the two values in the Figure 7d demonstrates that, as the Na^+^ levels increase, the Ca^2+^ and Mg^2+^ levels decrease, and cation exchange occurs. The R^2^ value was highest for the QLA aquifer, followed in descending order by the CFA and PFA aquifers. This result corresponds to the cation exchange strength. The ratio of Ca^2+^ and Mg^2+^ to SO_4_^2−^ in most of the water samples in Figure 7e is close to 1. This indicates that SO_4_^2−^ mainly comes from gypsum dissolution. The ratio of HCO_3_^−^ to Ca^2+^ in Figure 7f reflects carbonate dissolution and cation exchange.

**Figure 8:**
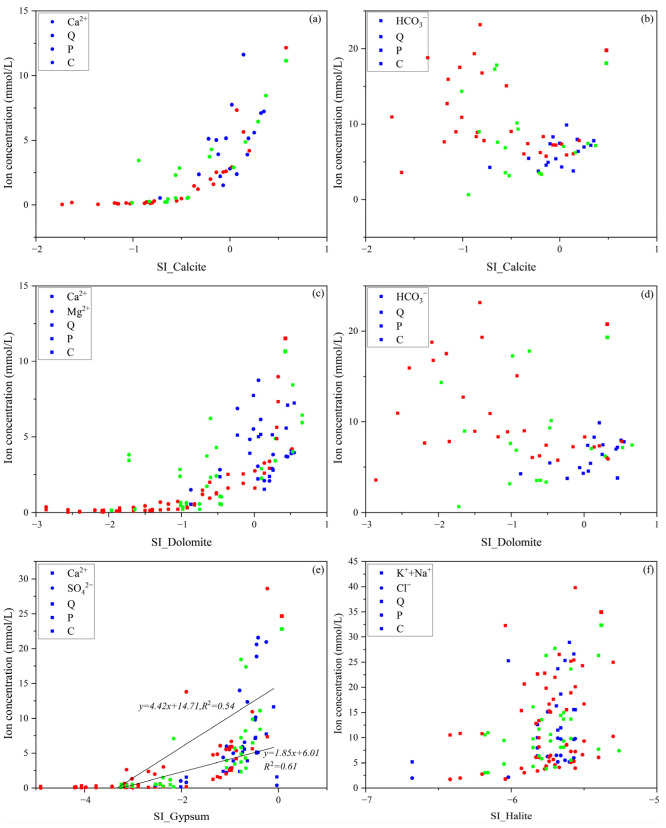
Scatter plots of major ions with respected carbonates minerals SI values: (**a**) Correlations of Ca^2+^ with respected calcite SI values, (**b**) Correlations of HCO_3_^−^ with respected calcite SI values, (**c**) Correlations of Ca^2+^ and Mg^2+^ with respected dolomite SI values, (**d**) Correlations of HCO_3_^−^ with respected dolomite SI values, (**e**) Correlations of Ca^2+^ and SO_4_^2^^−^ with respected gypsum SI values, (**f**) Correlations of K^+^+Na^+^ and Cl^−^ with respected halite SI values.

To further verify the source of ions in groundwater, the saturation index (SI) method is used to study the saturation state of minerals relative to groundwater. This helps to determine whether minerals have dissolved. Figure 8a–d shows that the saturation index values of calcite and dolomite in each aquifer are correlated with the increasing trend of Ca^2+^ and Mg^2+^ concentration. In contrast, there is a poor correlation between bicarbonate concentration and saturation index. Carbonate minerals with high SI values are related to a high bicarbonate concentration. The SI value of gypsum in Figure 8e has a correlation with the Ca^2+^ and SO_4_^2−^ concentrations. This may indicate that there is gypsum dissolution in each aquifer. The SI values in Figure 8f are less than 0, indicating that halite dissolution occurs in all aquifers. Comparing Figure 8a,b,d,e, it seems than samples with concentrations of Ca, Mg, and HCO_3_^−^ in the range of 1–8 (aprox.) mmol/L are the less modified by cation exchange, while water with HCO_3_^−^ concentrations >>8–10 mmol/L are the most affected by cation exchange.

#### 4.3.2. Analysis of Contribution Rate of Main Factors

The common factors across 67 groups of water samples from the Sunan mining area were extracted using SPSS software, generating three main factors defined in the following section, namely F_1_, F_2_, and F_3_. The total variance interpretation table (Table 3) was obtained using the maximum variance method. The eigenvalues of F_1_, F_2_, and F_3_ were 3.564, 4.541, and 1.087, respectively; the cumulative contribution rate was 88.455%, indicating that the three factors effectively explain most of the information related to the 7 variables.

**Figure 9:**
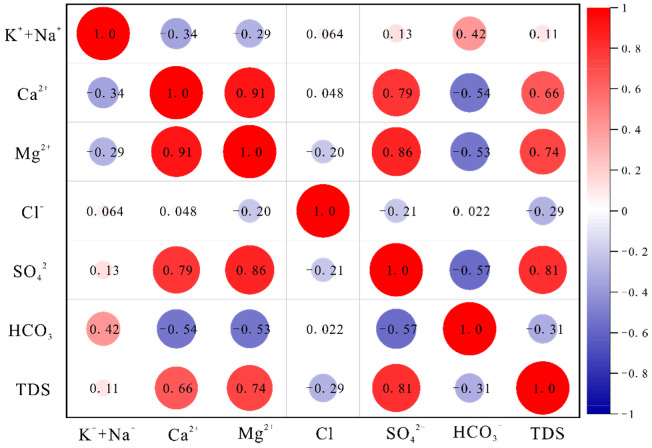
Comprehensive correlation heat plot.

Table 4 shows that the principal factor F_1_ takes Ca^2+^, Mg^2+^, SO_4_^2−^, and TDS as the main loads. The four variables are significantly correlated (Figure 9). This indicates that the dissolution and enrichment of gypsum and magnesium salt control the change in TDS levels during the natural migration of groundwater, while Ca^2+^, Mg^2+^, and K^+^ + Na^+^ are negatively correlated. This demonstrates there is also cation exchange in groundwater. Therefore, F_1_ is defined to be sulfate dissolution and cation exchange. The contribution rates of factor F_1_ to Ca^2+^, Mg^2+^, SO_4_^2−^, and TDS are 62.68%, 68.44%, 88.70%, and 68.86%, respectively.

The main load of factor F_2_ is K^+^ + Na^+^ and HCO_3_^−^. There is a positive correlation between them. This indicates that the dissolution of carbonate rock mainly occurs in the groundwater, accompanied by the dissolution of calcite and dolomite. Therefore, F_2_ can be defined to be the carbonate dissolution and sulfate reduction. The factor’s contribution rates to K^+^ + Na^+^ and HCO_3_^−^ are 89.44% and 49.31%, respectively.

The main load of factor F_3_ is Cl^−^, which is weakly correlated with other ions. Furthermore, Cl^−^ is mainly affected by mining and human activities to produce industrial and domestic sewage. Therefore, factor F_3_ is defined as the external input factor. The contribution of external input factor (F_3_) to Cl^−^ is 88.13%.

In conclusion, the contribution rates of F_1_, F_2_, and F_3_ to groundwater are 49.45%, 29.53%, and 21.01%, respectively. These results indicate that sulfate dissolution and cation exchange have the most significant impact on the groundwater aquifer.

**Figure 10:**
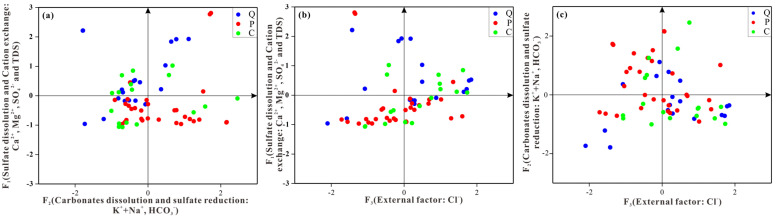
Scatter plots of principal factor scores: (**a**) F_1_ versus F_2_, (**b**) F_1_ versus F_3_, (**c**) F_2_ versus F_3_.

Combined with the three main extracted factors, the factor scores of each water sample were calculated using SPSS software. Figure 10 shows the scatter diagram of the main factor scores of each aquifer. The Quaternary water sample points are mainly distributed in the first and second quadrants of Figure 10a,b, and the fourth quadrant of Figure 10c. This shows that sulfate dissolution, cation exchange, carbonate dissolution, and the input of external factors occur in the aquifer. The Permian water samples are mainly distributed in the third and fourth quadrants of Figure 10a,b, and the second, third, and fourth quadrants of Figure 10c. This indicates that carbonate dissolution, sulfate reduction, cation exchange, and the input of external factors occur in the Permian aquifer. Carboniferous water samples are mainly distributed in the second and third quadrants of Figure 10a, the first and third quadrants of Figure 10b, and the third and fourth quadrants of Figure 10c. This indicates that sulfate dissolution, cation exchange, and the input of external factors occur in the Carboniferous aquifer.

**Figure 11:**
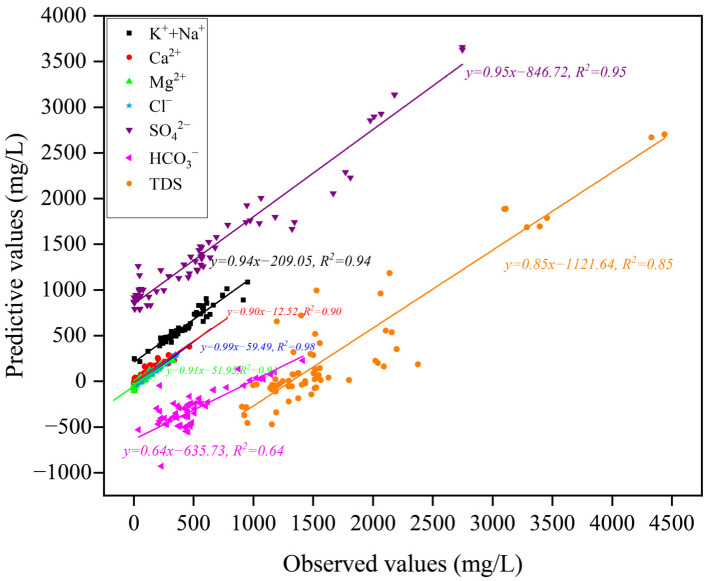
Scatter plots of predicted and observed concentration.

The APFS-MLR method was used to establish the functional relationship between the concentration of variables and absolute principal factor. Based on the functional model, the concentration of each variable was predicted, and the predicted value was compared with the measured value. Figure 11 shows that the R^2^ values of multiple linear regression of each variable are between 0.64 and 0.98. The HCO_3_^−^ fitting value is low, at 0.64, and the value associated with the other variables is close to 1. This indicates that HCO_3_^−^ may be unstable in the aquifer, with significant differences in concentrations. This further indicates that the APFS-MLR model is effective in calculating and determining the distribution of ion sources in groundwater.

#### 4.3.3. Spatial Distribution Characteristics of Principal Factors

We evaluated the effect of interpolation by percent variation (Equation (1)). The F factors with outlier values had higher PV values compared to elements with lower PV values (Table 5). However, the Kriging interpolation effect is worse for QFsum, PF2, PFsum, and CFsum, as it was influenced by the small number of samples.

**Figure 12:**
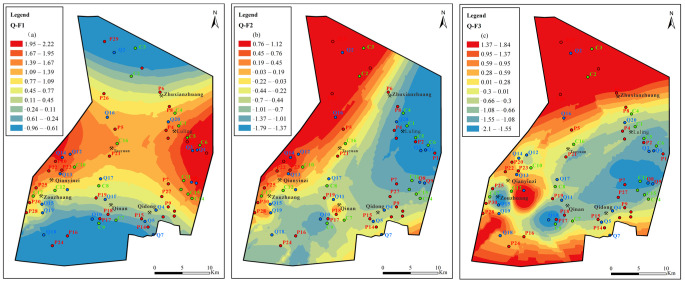
Contours of principal factors loading scores for Quaternary aquifer: (**a**) sulfate dissolution and cation exchange, (**b**) carbonate dissolution and sulfate reduction, (**c**) external input factor.

In the Quaternary aquifer (Figure 12), the areas with high F_1_ values are located on the east and west sides of the Sunan mining area; the F_1_ value is higher on the east side compared to the west side. This is because the Sunan syncline and anticline structures are located on the east and west sides. Generally, the two wings of the fold are recharged runoff areas, and there is strong sulfate rock dissolution and cation exchange. The area with the high value of F_1_ is mainly distributed in the Qianyingzi coal mine, Taoyuan Coal Mine and Luling coal mine, Zouzhuang coal mine, and Xunzhuang coal mine. The F_1_ factor scores in the Qidong Coal Mine, Qinan Coal Mine, and the northernmost part of the mining area are low and, in most areas, F1 values are negative.

The areas with high F_2_ values are mainly located in Qianyingzi coal mine and the north part of Taoyuan Coal Mine. Carbonate dissolution (calcite and dolomite dissolution) and sulfate reduction occur mainly in this area. The F_2_ scores of other coal mines are low and most of them are negative, indicating that F_2_ has a weak or no effect.

The areas with high F_3_ factor scores are mainly in the southern and northern ends of the mining area and near the Sunan anticline. This area does not belong to the coal mining area, and agricultural domestic water seeps into the underground water. This means that the F_3_ factor scores are mainly driven by external factors.

**Figure 13:**
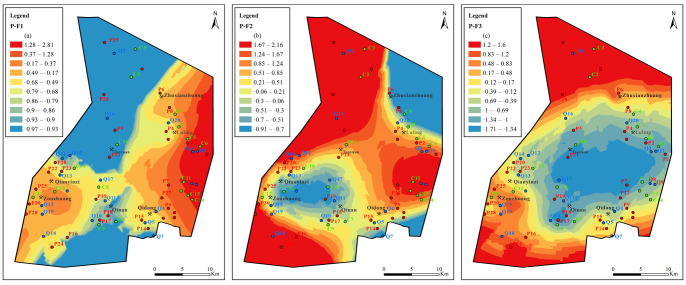
Contours of principal factors loading scores for Permian aquifer: (**a**) sulfate dissolution and cation exchange, (**b**) carbonate dissolution and sulfate reduction, (**c**) external input factor.

In the Permian aquifer (Figure 13), F_1_ factor scores gradually increase from west to east. Luling Mine is located in the area with high factor values, and some areas of Qianyingzi mine and Zouzhuang mine have positive scores. Sulfate dissolution and cation exchange are strong in these mining areas. This changes the underground aquifer from being an anaerobic environment to being an aerobic environment, resulting in pyrite oxidation. The Luling Mine, Zouzhuang mine, and the northern and southern parts of the mining area are all areas with high F_2_ values. The F_2_ scores in some areas of Qianyingzi mine and Taoyuan mine range from 0.21–0.85. Coal mining activities lead to an increase in carbonate dissolution (calcite and dolomite dissolution), which leads to sulfate reduction. Areas with high F_3_ factor scores are mainly located in the south and north of the mining area; these areas are affected by the domestic water of agricultural residents. Another area with a high F_3_ factor value appears in the Zouzhuang coal mine, which indicates that the area is affected by the industrial water generated by mining. These areas are greatly affected by external drivers.

**Figure 14:**
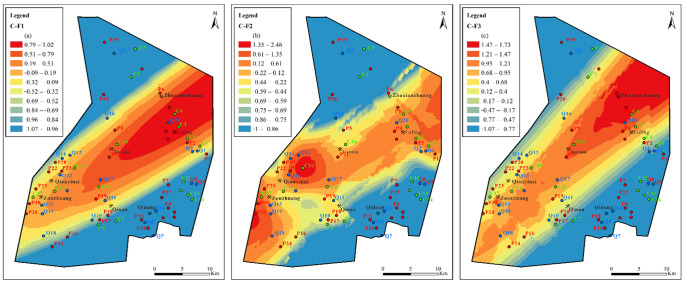
Contours of principal factors loading scores for Carboniferous aquifer: (**a**) sulfate dissolution and cation exchange, (**b**) carbonate dissolution and sulfate reduction, (**c**) external input factor.

In the Carboniferous aquifer (Figure 14), the high values of factors F_1_, F_2_, and F_3_ are all located in the coal mine concentration area; the values increase from southwest to northeast. Sulfate dissolution, carbonate dissolution, and cation exchange jointly control the hydrogeochemical process of the limestone aquifer.

**Figure 15:**
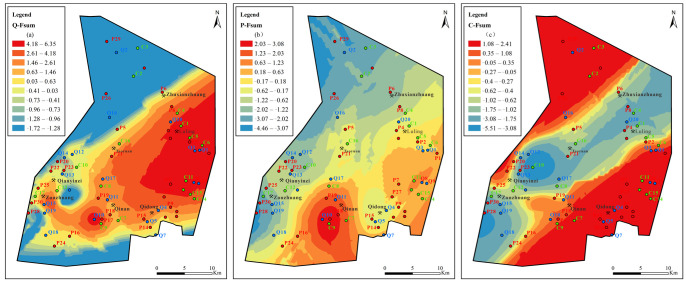
Contours of comprehensive principal factors loading scores for Quaternary, Permian, and Carboniferous aquifers: (**a**) sulfate dissolution and cation exchange, (**b**) carbonate dissolution and sulfate reduction, (**c**) external input factor.

The value (score) of comprehensive factors is calculated from the variance contribution rate of each factor in Table 2. Figure 15 shows that the comprehensive factor score is higher in the QLA water in the area from Zhuxianzhuang and Luling to the east wing of the Sunan inclined direction. The comprehensive value is higher in the PFA water near the Qilan coal mine, and a high score appears in the CFA water in Qilan, from Qidong to Zhuxianzhuang and Luling, and at the northern end of the extreme north of the mining area. Therefore, the Zhuxianzhuang mine, Luling mine, Qidong mine, and Qilan mine are the most driven by F_1_, F_2_, and F_3_. The groundwater in the Sunan mining area is affected by both the original geological environment and human activities. This makes the spatial distribution of the main controlling factors of each aquifer different and complex.

### 4.4. Inverse Modeling of Hydrogeochemical Processes in Multiple Aquifers

The inverse modeling mainly focuses on K^+^ + Na^+^, Ca^2+^, Mg^2+^, HCO_3_^−^, SO_4_^2−^, and Cl^−^ to conduct reverse hydrogeochemical process modeling. Initial and final water samples are selected to reflect and characterize the groundwater flow. Based on the previous theoretical analysis results, Table 6 lists the possible mineral phases and reactions occurring in the hydrogeochemical processes.

#### 4.4.1. Source and connection of Permian aquifer

Groundwater level observation data indicate that Permian aquifer may come from Cenozoic water and Carboniferous Ordovician limestone water. Using C13 and O2 as the initial water sample, and P7 as the final water sample, a model is generated with an uncertainty level of 0.05. Then, taking P27 as the final water sample, and Q9, C11, and O2 as the initial water sample, a model is generated with an uncertainty level that is also 0.05. The results are shown in Table 7. This indicates there may be two sources of Permian sandstone water.

Source 1 of the Permian sandstone water is explained by the fact that the water in P7 is related to C13 and O2, and the sum of the fractions of C13 and O2 is exactly 1. Combined with the spatial distribution of these water samples, P7 relates solely to C13 and O2. This indicates a strong hydraulic connection with the deep limestone aquifer. In addition, the hydrogeochemical process in Path 1 includes halite dissolution and cation exchange. This may explain why K^+^ + Na^+^ is higher in P7 compared to C13 and O2, while Ca^2+^ and Mg^2+^ are lower compared to C13 and O2. The molar value of gypsum is negative, indicating that sulfate reduction may have occurred. This may also explain why SO_4_^2−^ is lower in P7 compared to C13 and O2.

Source 2 of the Permian sandstone water is explained by the fact that the water in P27 is related to Q9, C11, and O2. The sum of the fractions of C11 and Q9 is 1.086, which indicates that connection occurs between Carboniferous and loose water. The sum of the fractions of C11 and O2 is 1.577, which also demonstrates that there is a connection between Ordovician aquifers. As groundwater flows, gypsum dissolution and calcite precipitation occur, somewhat maintaining the stability of calcium ion concentration in P27 water.

#### 4.4.2. Source and Connection of Carboniferous Aquifer

Based on the spatial distribution of the water sample points, C10 and C15 water samples on both sides of Xisipo branch fault are selected. This was carried out to test the hypothesis that C15 water samples may be from C10 and Q8 water. Table 8 shows the results of the reverse hydrological modeling. The fractions of C10 and Q8 add up to 1, demonstrating that C15 does indeed relate solely to C10 and Q8. The calcite dissolution, dolomite precipitation, and the halite precipitation result in K^+^ + Na^+^ being supersaturated. The Ca^2+^ and Mg^2+^ in the C15 water are present at a lower level compared to C10 and Q8. This shows that cation exchange occurs in the solution, and that K^+^ + Na^+^ in the rock are exchanged with calcium and magnesium ions in water. The results show that the calcium magnesium ion levels decrease as a result.

#### 4.4.3. Source and Connection of Ordovician Aquifer

To identify the source and hydraulic connection of the Ordovician limestone water, water samples C12, Q6, and O1, collected near the Shuangdui fault, were used for a simulation. The results are shown in Table 9. The sum of the fractions of C12 and Q6 is exactly 1, demonstrating that O1 is only related to C12 and Q6. This shows that the Ordovician water mainly comes from Carboniferous water and loose water, and calcite dissolution occurs during groundwater recharge, dolomite precipitation, and cation exchange. Cation exchange causes the decrease in Ca^2+^ and Mg^2^ ion levels in O1 compared with C12 and Q6.

#### 4.4.4. Main Hydrogeochemical Paths of Groundwater System with Multiple Aquifers

**Figure 16:**
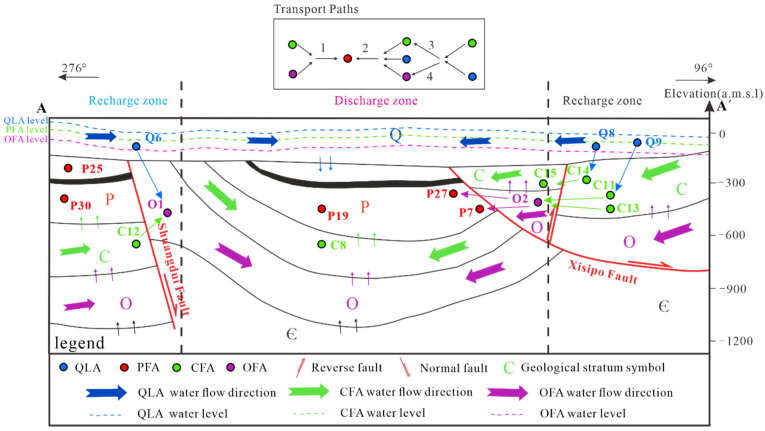
Hydrogeochemical path of multi-layer groundwater system.

Figure 16 shows that four major hydrogeochemical pathways are identified. Hydrogeochemical processes occur within the same aquifer, and in the connection between aquifers.

Paths 1 and 2 show the main source of the Permian coal measure sandstone water. The hydrogeochemical simulation results indicate that the water may come from QLA, CFA, and OFA, and that it is mainly accompanied by halite dissolution, dolomite dissolution, gypsum dissolution, and calcite precipitation. If the mine is closed in the future, and if the water level rises to the top of the mine, the QLA may be recharged, polluting the aquifer. Measures should be taken to prevent the pollution of the QLA caused by a possible future water level rise.

Path 3 shows the connection of Carboniferous limestone aquifers. The simulation results show a connection between CFA on both sides of the Shuangdui fault. The CFA lies in the footwall of the fault supplying hanging wall aquifers, while QLA also supplies hanging wall limestone aquifers. This process mainly involves calcite dissolution, dolomite precipitation, and cation exchange. The coal seam mining floor is likely to produce cracks, which may lead to limestone aquifer recharge of PFA. If the mine is closed in the future, and the mine water is supplemented, PFA may be mixed with limestone water and loose water to create cross-pollution. This highlights the importance of frequently monitoring the water level and water quality changes, as well as implementing measures that prevent the cross-pollution of the aquifer.

Path 4 shows the source and connection of the Ordovician limestone aquifers. The simulation results and hydrogeological conditions indicate that shallow water from the QLA and CFA aquifers mainly supplies the Ordovician aquifers through the Xisipo fault. They are mainly affected by calcite dissolution, dolomite precipitation, and cation exchange. Again, this highlights the need to prevent the pollution of industrial and domestic water.

## 5. Conclusions

This study focused on the Sunan mine area affected by mining activities in the east area of the Huaibei Coalfield, and identified the hydrogeochemical process and migration path of the multi-layer aquifer system using the conventional analysis method of hydrochemistry, FA-GIS, the APFS-MLR method, and reverse hydrogeochemical modeling. The investigation yielded the following conclusions:

1. The pH value of the entire aquifer system is weakly alkaline. The QLA included SO_4_−Na, HCO_3_−Na, and HCO_3_−Ca hydrochemical water types; the PFA includes the HCO_3_−Na water type; and the CFA includes the HCO_3_−Na and SO_4_−Na water types. The hydrochemical types of these aquifers are similar, indicating the presence of hydraulic connections;
2. The main hydrogeochemical processes of the groundwater system include dissolution and precipitation of minerals, sulfate reduction, and cation exchange. The main reactants include calcite, dolomite, gypsum, halite, organic carbon, O_2_, CO_2_, and water;
3. Using the FA/APFS-MLR model, the average contribution rates of the sulfate dissolution factor and cation exchange factor (F1), carbonate dissolution factor and sulfate reduction factor (F2), and external input factor (F3) to the groundwater hydrochemical index are determined as 49.45%, 29.53%, and 21.01%, respectively;
4. The PFA water is mainly supplied by the overlying QLA and underlying limestone water through fractures and faults. This water movement is accompanied by mineral dissolution and precipitation, cation exchange, and sulfate reduction;
5. The Carboniferous and Ordovician karst aquifers connect with each other and supply the Permian aquifers through faults and fractures. In addition, the Quaternary aquifers supply the karst aquifers through fractures. It is important to pay attention to the possible cross-pollution that could be caused by the mixture of Permian mine water and karst aquifers. Human industrial activities should be prevented from polluting the Quaternary loose water, and also from aggravating the pollution of karst aquifers.

## Figures and Tables

**Table 1:** Measured values of major ions of the water samples in the study area.

Sample ID	K^+^ + Na^+^	Ca^2+^	Mg^2+^	Cl^−^	SO_4_^2−^	HCO_3_^−^	PH	TDS	Percent Error (%)	Sample ID	K^+^ + Na^+^	Ca^2+^	Mg^2+^	Cl^−^	SO_4_^2−^	HCO_3_^−^	PH	TDS	Percent Error (%)
Q1	5.5	464.92	330.62	49.08	2179.6	230.56	7.58	3098	−1.11	P16	456.21	5.54	3.84	155.22	189.34	466.23	9.44	1188	4.61
Q2	5.3	464.62	330.69	49.27	2180.4	230.63	7.59	3106	−1.18	P17	241.80	8.73	17.32	59.94	44.86	541.86	8.78	1799.00	−4.31
Q3	5.26	464.58	330.67	49.27	2179.4	230.83	7.25	3109	−1.16	P18	248.93	19.79	13.44	68.79	57.21	549.32	7.96	1513.00	−4.49
Q4	347.58	88.58	73.31	221.47	572.12	451.35	7.55	1515	−3.39	P19	248.31	8.73	13.47	97.01	0.41	507.78	8.49	1377.00	−2.21
Q5	307.35	103.54	78.43	216.24	560.19	440.48	7.71	1518	−3.44	P20	353.37	79.99	22.57	138.66	531.38	380.36	7.97	1329	−3.35
Q6	288.93	111.41	91.74	226.57	567.6	454.93	8.02	1337	−3.49	P21	584.73	6.34	0.96	188.18	46.92	1022.75	9.13	1436	−1.75
Q7	374.53	95.04	49.95	230.44	420.24	602.9	7.76	1547	−4.44	P22	579.03	1.58	2.88	181.58	16.88	1145.31	8.92	1406	−5.43
Q8	274.99	205.47	69.41	344	567.53	389.48	7.92	1563	−2.74	P23	610.26	4.76	4.81	145.26	60.09	1179.12	9.38	1504	−3.23
Q9	264.18	223.18	88.82	350.88	628.03	425.42	7.41	1535	−2.88	P24	229.03	167.53	100.68	289.46	514.5	477.42	8.31	1142.00	−3.82
Q10	49.82	21.57	35.75	17.88	36.22	258.72	8.3	1486	−2.04	P25	434.56	101.34	46.02	257.36	637.57	441.62	8.46	1377.00	−3.12
Q11	581.3	94.34	67.63	74.89	1343.46	333.16	8.6	1402	−1.88	P26	462.32	58.73	28.68	256.09	582	368.27	8.12	1297.00	−2.66
Q12	544.09	200.38	209.88	184.36	1976.91	277.14	7.62	3287	−1.2	P27	357.47	225.25	116.96	304.26	1049.58	369.78	8.77	1335.00	−2.34
Q13	582.38	309.68	132.55	194.99	2010.25	262.55	7.44	3395	−1.09	P28	779.68	4.79	3.39	215.38	6.17	1412.39	8.73	1027	0.01
Q14	665.09	204.33	165.2	184.36	2069.94	228.52	8.54	3457	−0.17	P29	915.35	7.21	8.75	139.77	1324.94	217.54	8.7	1197	6.83
Q15	119.42	60.91	50.5	68.03	76.97	505.25	8.22	1526	−5.06	P30	779.68	4.79	3.39	215.38	6.17	1412.39	8.44	999	0.01
Q16	357.21	289.27	94.83	347.08	973.02	474.89	7.83	1480	−2.71	C1	252.68	20.95	24.62	107.69	47.75	617.88	7.93	942	−7.8
Q17	229.61	155.54	98.87	283.43	477.46	485.72	8.33	1433	−3.79	C2	242.33	22.88	25.4	106.83	52.68	568.83	8.35	935	−6.03
Q18	357.74	283.82	88.23	341.22	940.09	438.28	7.55	1502	−1.53	C3	314.75	137.55	91.64	347.47	544.89	40.39	7.65	1320.68	4.9
Q19	429.66	156.33	115.77	229.27	1183.76	300.73	7.71	1518.00	−1.81	C4	244.44	194.93	82.08	330	556.35	378.81	7.23	1630.4	−2.76
Q20	612.38	206.09	147.47	211.96	1812.27	330.09	7.27	1528	−1.54	C5	637.39	92.17	149.09	147.14	1770.6	216.17	7.44	2139.883	−0.99
P1	742.44	293.06	215.67	61.92	2747.64	360.26	7.6	4438	−1.48	C6	605.81	113.49	57.16	135.17	1667.17	192.27	7.2	2065.036	−5.15
P2	742.44	293.06	215.67	61.92	2747.64	360.26	7.7	4328	−1.48	C7	268.92	21.75	15.14	225.43	8.23	418.42	7.16	951.35	−3.01
P3	520.28	3.05	1.07	119.2	125.1	971.3	8.49	1222	−5.28	C8	369.98	10.29	4.8	152.45	140.36	547.01	7.14	904.5	−3.32
P4	474.74	1.18	4.31	106.78	251.9	667.84	8.62	1246	−1.29	C9	311.44	18.21	15.36	206.37	113.19	463.92	7.5	925.32	−5.32
P5	505.63	3.89	8.02	163.09	227.2	775.66	8.5	2041	−4.25	C10	951.63	6.57	3.43	263.25	679.55	874.18	9.18	1557	3.81
P6	351.83	64.07	65.82	218.04	465.93	507.44	5.59	1157	−4.52	C11	216.68	115.65	54.17	168.74	377.44	428.81	7.8	1157	−4.17
P7	574.15	12.44	8.15	363.22	2.88	919.37	8.44	1192	−4.46	C12	314.87	337.47	96.25	329.28	1064.4	436.34	8.1	2108.139	−2.66
P8	383.25	12.16	2.74	329.64	4.53	476.44	8.43	1156	−3.91	C13	224.02	257.48	142.49	285.97	946.27	453.47	8.55	2158.53	−4.45
P9	405.17	48.9	30.88	200.94	456.88	452.29	8.55	1185	−3.69	C14	331.09	171.63	57.77	286.46	787.39	204.15	7.46	2198.355	−1.7
P10	307.35	103.54	78.43	216.24	560.19	440.48	8.55	1185	−3.5	C15	344.29	149.28	41.42	287.88	690.66	214.11	7.01	2021.338	−2.01
P11	294.4	116.67	81.14	216.47	568.83	448.02	10.96	1557	−4.03	C16	605.57	8.64	8.58	272.77	36.63	1053.15	6.88	2376.129	−3.59
P12	346.31	100.55	38.41	229.3	528.91	350.83	8.55	1198	−2.9	C17	542.78	8.71	13.44	195.79	47.33	1084.8	6.97	2091.107	−5.77
P13	523.85	3.50	2.23	133.17	11.52	1068.50	8.54	1295.00	−4.62	O1	207.20	152.06	66.72	216.83	490.06	337.18	7.3	1045.07	−2.43
P14	382.86	5.53	10.53	187.81	21.81	545.92	7.7	1297.00	3.33	O2	211.84	148.51	70.3	126.32	491.76	333.67	7.97	1043.26	5.22
P15	558.19	6.73	16.18	234.59	291.41	664.73	8.61	1513.00	−0.04										

**Table 2:** Statistics of all water samples.

Aquifers	Statistics	K^+^ + Na^+^	Ca^2+^	Mg^2+^	Cl^−^	SO_4_^2−^	HCO_3_^−^	pH	TDS
QLA	Mean	320.12	210.18	132.52	193.73	1137.77	367.58	7.81	2017.1
	Median	327.47	202.36	93.29	214.1	956.56	361.32	7.71	1527
	Standard deviation	209.66	134.28	94.86	109.92	761.25	112.92	0.4	828.41
	Skewness	0.05	0.82	1.41	0.14	0.23	0.27	0.63	0.97
	Min.	5.26	21.57	35.75	17.88	36.22	228.52	7.25	1337
	Max.	665.09	464.92	330.69	350.88	2180.4	602.9	8.6	3457
PFA	Mean	479.79	59.11	38.88	185.16	469.35	650.05	8.48	1531.2
	Median	459.27	10.45	13.46	188	239.55	507.61	8.52	1297
	Standard deviation	180.76	84.68	57.47	79.91	700.9	332.34	0.83	804.39
	Skewness	0.64	1.78	2.2	0.25	2.5	1.06	0.58	3.25
	Min.	229.03	1.18	0.96	59.94	0.41	217.54	5.59	999
	Max.	915.35	293.06	215.67	363.22	2747.64	1412.39	10.96	4438
CFA	Mean	398.75	99.27	51.93	226.39	560.64	481.92	7.62	1616.57
	Median	314.87	92.17	41.42	225.43	544.89	436.34	7.46	1630.4
	Standard deviation	201.44	99.41	46.33	80.45	556.12	295.06	0.63	557.01
	Skewness	1.53	1.01	0.96	0.07	1.01	0.82	1.09	0.17
	Min.	216.68	6.57	3.43	106.83	8.23	40.39	6.88	904.5
	Max.	951.63	337.47	149.09	347.47	1770.6	1084.8	9.18	2376.13

**Table 3:** Total variance explained.

Component	Extraction Sums of Squared Loadings		
	Total	% Of Variance	Cumulative/%
F_1_	3.564	50.914	50.914
F_2_	1.541	22.011	72.926
F_3_	1.087	15.529	88.455

**Table 4:** Rotating component matrix and contribution rate of principal factors.

Variables	Principal Factors			Contribution Rate (%)		
	F_1_	F_2_	F_3_	F_1_	F_2_	F_3_
K^+^ + Na^+^	0.05	**0.97**	0.07	4.43	**89.44**	6.13
Ca^2+^	**0.87**	−0.37	0.15	**62.68**	26.44	10.88
Mg^2+^	**0.90**	−0.31	−0.11	**68.44**	23.37	8.19
Cl^−^	−0.11	0.03	**0.99**	9.35	2.52	**88.13**
SO_4_^2−^	**0.97**	0.04	−0.09	**88.70**	3.48	7.83
HCO_3_^−^	−0.53	**0.60**	−0.08	43.72	**49.31**	6.97
TDS	**0.88**	0.16	−0.24	**68.86**	12.21	18.94

Significant factors are in bold.

**Table 5:** Statistical results of the percentage of variation (PV) calculated between the values measured in the laboratory and those estimated by the interpolation applied to the sampling points, for all groundwater samples.

	QF1	QF2	QF3	QFsum	PF1	PF2	PF3	PFsum	CF1	CF2	CF3	CFsum
min	0.00	0.00	0.01	0.03	0.03	0.09	0.03	0.01	0.14	0.02	0.08	0.05
max	10.07	12.63	2.02	44.28	8.02	61.78	10.99	18.62	2.09	1.87	10.04	72.02
mean	1.33	1.29	0.72	3.54	1.33	3.18	1.75	2.31	0.99	0.98	1.81	6.18
median	0.61	0.32	0.70	1.08	0.42	0.63	0.67	0.89	1.06	1.16	0.64	0.64

**Table 6:** Potential hydrogeochemical reaction phase.

Phase	Formula	Reaction
Calcite	CaCO_3_	CaCO_3_ = Ca^2+^ + CO_3_^2^–
Dolomite	CaMg(CO_3_)_2_	CaMg(CO_3_)_2_ = Ca^2+^ + Mg^2+^ + 2CO_3_^2–^
Gypsum	CaSO_4_·2H_2_O	CaSO_4_·2H_2_O = Ca^2+^ + SO_4_^2−^+ 2H_2_O
Halite	(Na + K)Cl	(Na + K)Cl = (Na + K)^+^ + Cl^−^
NaX	NaX	NaX = Na^+^ + X^−^
CaX_2_	CaX_2_	CaX_2_= Ca^2+^ + 2X^−^
MgX_2_	MgX_2_	MgX_2_= Mg^2+^ + 2X^−^
H_2_O(g)	H_2_O(g)	H_2_O(g) = H_2_O(a)
CO_2_(g)	CO_2_(g)	CO_2_(g) = CO_2_(a)
O_2_(g)	O_2_(g)	O_2_(g) = O_2_(a)

**Table 7:** Result for the inverse geochemical modeling of Permian fractured aquifer connections.

Water Sample and Phases	Formula	Flow and Mixing Path:	Indications
Origin 1		C13/O2→P7	
		Fractions and mole transfer (mol/L)	
C13	-	0.620	
O1	-	0.370	
P7	-	1	
CO_2_ (g)	CO_2_ (g)	5.41 × 10^−3^	
Gypsum	CaSO_4_·2H_2_O	−7.67 × 10^−3^	
Halit	(Na + K)Cl	2.66 × 10^−3^	
MgX_2_	MgX_2_	−6.88 × 10^−3^	
NaX	NaX	1.38 × 10^−2^	
Dolomite	CaMg(CO_3_)_2_	2.32 × 10^−3^	
Origin 2		Q9/C11/O2 → P27	
		Fractions and mole transfer (mol/L)	
Q9	-	0.018	
C11	-	1.068	
O2	-	0.499	
P27	-	1	
Calcite	CaCO_3_	−2.244 × 10^−3^	
CO_2_ (g)	CO_2_ (g)	−3.008 × 10^−3^	
Gypsum	CaSO_4_·2H_2_O	1.431 × 10^−3^	
H_2_O (g)	H_2_O (g)	−32.54	

**Table 8:** Result for the inverse geochemical modeling of Carboniferous fractured limestone aquifer connections.

Water Sample and Phases	Formula	Flow and Mixing Path:	Indications
		C10/Q8 → C15	
		Fractions and mole transfer (mol/L)	
C10	-	0.670	
Q8	-	0.320	
C15	-	1	
Calcite	CaCO_3_	1.161 ×10^−3^	
NaX	NaX	2.386 × 10^−3^	
MgX_2_	MgX_2_	−4.88 × 10^−3^	
CO_2_(g)	CO_2_(g)	1.072 × 10^−4^	
Dolomite	CaMg(CO_3_)_2_	−8.275 × 10^−4^	
Halite	(Na + K)Cl	−4.815 × 10^−4^	

**Table 9:** Result for the inverse geochemical modeling of Ordovician fractured limestone aquifer connections.

Water Sample and Phases	Formula	Flow and Mixing Path:	Indications
		C12/Q6 → O1	
		Fractions and mole transfer (mol/L)	
C12	-	0.572	
Q6	-	0.428	
O1	-	1	
Calcite	CaCO_3_	1.229 × 10^−3^	
Gypsum	CaSO_4_·2H_2_O	−3.847 × 10^−3^	
CO_2_ (g)	CO_2_(g)	−4.689 × 10^−4^	
Dolomite	CaMg(CO_3_)_2_	−1.089 × 10^−3^	
Halite	(Na + K)Cl	−3.447 × 10^−3^	

## Data Availability

Not applicable.

## References

[1] Bi Y., Wu J., Zhai X., Wang G., Shen S., Qing X. (2021). Discriminant Analysis of Mine Water Inrush Sources with Multi-Aquifer Based on Multivariate Statistical Analysis. Environ. Earth Sci..

[2] Zhang H., Xing H., Yao D., Liu L., Xue D., Guo F. (2019). The Multiple Logistic Regression Recognition Model for Mine Water Inrush Source Based on Cluster Analysis. Environ. Earth Sci..

[3] Adhikari K., Mal U. (2019). Application of Multivariate Statistics in the Analysis of Groundwater Geochemistry in and around the Open Cast Coal Mines of Barjora Block, Bankura District, West Bengal, India. Environ. Earth Sci..

[4] Bi Y., Wu J., Zhai X., Shen S., Tang L., Huang K., Zhang D. (2021). Application of Partial Least Squares-Discriminate Analysis Model Based on Water Chemical Compositions in Identifying Water Inrush Sources from Multiple Aquifers in Mines. Geofluids.

[5] Ju Q., Hu Y. (2021). Source Identification of Mine Water Inrush Based on Principal Component Analysis and Grey Situation Decision. Environ. Earth Sci..

[6] Qian J., Tong Y., Ma L., Zhao W., Zhang R., He X. (2018). Hydrochemical Characteristics and Groundwater Source Identification of a Multiple Aquifer System in a Coal Mine. Mine Water Environ..

[7] Yang S., Ge M., Li X., Pan C. (2020). The Spatial Distribution of the Normal Reference Values of the Activated Partial Thromboplastin Time Based on ArcGIS and GeoDA. Int. J. Biometeorol..

[8] Gharaat M.J., Mohammadi Z., Rezanezhad F. (2020). Distribution and Origin of Potentially Toxic Elements in a Multi-Aquifer System. Environ. Sci. Pollut. Res..

[9] Huang P., Han S. (2017). Study of Multi-Aquifer Groundwater Interaction in a Coal Mining Area in China Using Stable Isotopes and Major-Ion Chemical Data. Environ. Earth Sci..

[10] Ju Q., Liu Y., Hu Y., Wang Y., Liu Q., Wang Z. (2020). Hydrogeochemical Evolution and Control Mechanism of Underground Multiaquifer System in Coal Mine Area. Geofluids.

[11] Qiao W., Li W., Zhang S., Niu Y. (2019). Effects of Coal Mining on the Evolution of Groundwater Hydrogeochemistry. Hydrogeol. J..

[12] Yin L., Ma K., Chen J., Xue Y., Wang Z., Cui B. (2019). Mechanical Model on Water Inrush Assessment Related to Deep Mining Above Multiple Aquifers. Mine Water Environ..

[13] Zhang H., Xu G., Chen X., Wei J., Yu S., Yang T. (2019). Hydrogeochemical Characteristics and Groundwater Inrush Source Identification for a Multi-aquifer System in a Coal Mine. Acta Geol. Sin.-Engl. Ed..

[14] Li X., Tang C., Cao Y., Li D. (2020). A Multiple Isotope (H, O, N, C and S) Approach to Elucidate the Hydrochemical Evolution of Shallow Groundwater in a Rapidly Urbanized Area of the Pearl River Delta, China. Sci. Total Environ..

[15] Gao Y., Qian H., Ren W., Wang H., Liu F., Yang F. (2020). Hydrogeochemical Characterization and Quality Assessment of Groundwater Based on Integrated-Weight Water Quality Index in a Concentrated Urban Area. J. Clean. Prod..

[16] Guo X., Zuo R., Wang J., Meng L., Teng Y., Shi R., Gao X., Ding F. (2019). Hydrogeochemical Evolution of Interaction Between Surface Water and Groundwater Affected by Exploitation. Groundwater.

[17] Zhao L., Ren T., Wang N. (2017). Groundwater Impact of Open Cut Coal Mine and an Assessment Methodology: A Case Study in NSW. Int. J. Min. Sci. Technol..

[18] Xie P., Li W., Yang D., Jiao J. (2018). Hydrogeological Model for Groundwater Prediction in the Shennan Mining Area, China. Mine Water Environ..

[19] Cui F., Wu L., Wu Q., Xiong C., Jin C., Li N., Liu D. (2020). Formation Mechanism of a Disastrous Groundwater Inrush Occurred at the Xinjing Coal Mine in Datong, Shanxi Province, China. Geomat. Nat. Hazards Risk.

[20] Elena F., Vasiliy L., Natalia K., Arslan S., Elena M., Ekaterina B., Anna K., Alexey M., Elena B. (2020). Hydrogeology and Hydrogeochemistry of Mineral Sparkling Groundwater within Essentuki Area (Caucasian Mineral Water Region). Environ. Earth Sci..

[21] Zabala M.E., Gorocito M., Dietrich S., Varni M., Murillo R.S., Manzano M., Ceballos E. (2021). Key Hydrological Processes in the Del Azul Creek Basin, Sub-Humid Pampean Plain. Sci. Total Environ..

[22] Qian J., Peng Y., Zhao W., Ma L., He X., Lu Y. (2018). Hydrochemical Processes and Evolution of Karst Groundwater in the Northeastern Huaibei Plain, China. Hydrogeol. J..

[23] Mussa K.R., Mjemah I.C., Muzuka A.N.N. (2020). A Review on the State of Knowledge, Conceptual and Theoretical Contentions of Major Theories and Principles Governing Groundwater Flow Modeling. Appl. Water Sci..

[24] Telahigue F., Souid F., Agoubi B., Chahlaoui A., Kharroubi A. (2020). Hydrogeochemical and Isotopic Evidence of Groundwater Salinization in a Coastal Aquifer: A Case Study in Jerba Island, Southeastern Tunisia. Phys. Chem. Earth Parts A/B/C.

[25] Kareem A., Mustafa O., Merkel B. (2018). Geochemical and Environmental Investigation of the Water Resources of the Tanjero Area, Kurdistan Region, Iraq. Arab. J. Geosci..

[26] Feng H., Zhou J., Chai B., Zhou A., Li J., Zhu H., Chen H., Su D. (2020). Groundwater Environmental Risk Assessment of Abandoned Coal Mine in Each Phase of the Mine Life Cycle: A Case Study of Hongshan Coal Mine, North China. Environ. Sci. Pollut. Res..

[27] Li P., Wu J., Tian R., He S., He X., Xue C., Zhang K. (2018). Geochemistry, Hydraulic Connectivity and Quality Appraisal of Multilayered Groundwater in the Hongdunzi Coal Mine, Northwest China. Mine Water Environ..

[28] Dong S., Zheng L., Tang S., Shi P. (2020). A Scientometric Analysis of Trends in Coal Mine Water Inrush Prevention and Control for the Period 2000–2019. Mine Water Environ..

[29] Xu K., Dai G., Duan Z., Xue X. (2018). Hydrogeochemical Evolution of an Ordovician Limestone Aquifer Influenced by Coal Mining: A Case Study in the Hancheng Mining Area, China. Mine Water Environ..

[30] Fernandes A.L., Cruz J.V., Figueira C., Prada S. (2020). Groundwater Chemistry in Madeira Island (Portugal): Main Processes and Contribution to the Hydrogeological Conceptual Model. Environ. Earth Sci..

[31] Luo M., Chen Z., Criss R.E., Zhou H., Huang H., Han Z., Shi T. (2016). Dynamics and Anthropogenic Impacts of Multiple Karst Flow Systems in a Mountainous Area of South China. Hydrogeol. J..

[32] Yuan J., Xu F., Deng G., Tang Y. (2018). Using Stable Isotopes and Major Ions to Identify Hydrogeochemical Characteristics of Karst Groundwater in Xide Country, Sichuan Province. Carbonates Evaporites.

[33] Liang S., Guo J., Wu P., Feng Y., Wang X., Wang G., Xu W., Luo Y., Wan L. (2020). Hydrogeochemical and Isotopic Characteristics of Surface Water and Groundwater in the Qinghai Lake Catchment (China). Arab. J. Geosci..

[34] Qu S., Shi Z., Liang X., Wang G., Han J. (2021). Multiple Factors Control Groundwater Chemistry and Quality of Multi-Layer Groundwater System in Northwest China Coalfield—Using Self-Organizing Maps (SOM). J. Geochem. Explor..

[35] Chen L., Feng X., Xu D., Zeng W., Zheng Z. (2018). Prediction of Water Inrush Areas Under an Unconsolidated, Confined Aquifer: The Application of Multi-Information Superposition Based on GIS and AHP in the Qidong Coal Mine, China. Mine Water Environ..

[36] Chen L., Gui H., Yin X. (2011). Monitoring of Flow Field Based on Stable Isotope Geochemical Characteristics in Deep Groundwater. Environ. Monit. Assess..

[37] Wang Q., Su X., Su L., Zhou F. (2020). CBM Geological Characteristics and Exploration Potential in the Sunan Syncline Block, Southern North China Basin. J. Pet. Sci. Eng..

[38] Ju Q., Hu Y., Liu Q., Liu Y., Hu T. (2022). Key Hydrological Process of a Multiple Aquifer Flow System in the Mining Area of Huaibei Plain, Eastern China. Appl. Geochem..

[39] Zhang J., Chen L., Hou X., Li J., Ren X., Lin M., Zhang M., Wang Y., Tian Y. (2022). Effects of Multi-Factors on the Spatiotemporal Variations of Deep Confined Groundwater in Coal Mining Regions, North China. Sci. Total Environ..

[40] Zhang M., Li C., Ma X., Yang L., Ding S. (2021). Evaluating the Mercury Distribution and Release Risks in Sediments Using High-Resolution Technology in Nansi Lake, China. J. Soils Sediments.

[41] Zhang J., Chen L., Li J., Chen Y., Ren X., Shi X. (2021). Analysis of Mining Effects on the Geochemical Evolution of Groundwater, Huaibei Coalfield, China. Environ. Earth Sci..

[42] Zhang M., Chen L., Yao D., Hou X., Zhang J., Qin H., Ren X., Zheng X. (2022). Hydrogeochemical Processes and Inverse Modeling for a Multilayer Aquifer System in the Yuaner Coal Mine, Huaibei Coalfield, China. Mine Water Environ..

[43] Zhang J., Chen L., Hou X., Ren X., Li J., Chen Y. (2022). Hydrogeochemical Processes of Carboniferous Limestone Groundwater in the Yangzhuang Coal Mine, Huaibei Coalfield, China. Mine Water Environ..

[44] Huang P., Yang Z., Wang X., Ding F. (2019). Research on Piper-PCA-Bayes-LOOCV Discrimination Model of Water Inrush Source in Mines. Arab. J. Geosci..

[45] Zhang H., Xu G., Chen X., Mabaire A., Zhou J., Zhang Y., Zhang G., Zhu L. (2020). Groundwater Hydrogeochemical Processes and the Connectivity of Multilayer Aquifers in a Coal Mine with Karst Collapse Columns. Mine Water Environ..

[46] Thurston G., Spengler J. (1985). A quantitative assessment of source contributions to inhalable particulate pollution in Metropolitan Boston. Atmos. Environ..

